# Comparative observation of mitochondrial morphology in *Arabidopsis* mutants of 4 reported fission factors

**DOI:** 10.1093/plphys/kiag531

**Published:** 2026-07-30

**Authors:** Masaru Hashimoto, Yugo Ito, Issei Nakazato, Hideki Takanashi, Shin-ichi Arimura

**Affiliations:** Graduate School of Agricultural and Life Sciences, The University of Tokyo, 1-1-1 Yayoi, Bunkyo-ku, Tokyo 113-8657, Japan; Graduate School of Agricultural and Life Sciences, The University of Tokyo, 1-1-1 Yayoi, Bunkyo-ku, Tokyo 113-8657, Japan; Graduate School of Agricultural and Life Sciences, The University of Tokyo, 1-1-1 Yayoi, Bunkyo-ku, Tokyo 113-8657, Japan; Graduate School of Agricultural and Life Sciences, The University of Tokyo, 1-1-1 Yayoi, Bunkyo-ku, Tokyo 113-8657, Japan; Graduate School of Agricultural and Life Sciences, The University of Tokyo, 1-1-1 Yayoi, Bunkyo-ku, Tokyo 113-8657, Japan

Dear Editor,

Mitochondria, which are not synthesized de novo, proliferate through growth and fission of preexisting ones (Kuroiwa et al. 1998), and the balance between fission and fusion determines their morphology (Arimura et al. 2004b). In *Arabidopsis thaliana* (hereafter *Arabidopsis*), 4 factors are reported to be implicated in mitochondrial fission: Dynamin-related protein 3 (DRP3), Fission 1 (FIS1), Elongated mitochondria 1 (ELM1), Peroxisomal and mitochondrial division factor (PMD), each with 2 paralogs in the genome: *DRP3A/3B*, *FIS1A*/*B*, *ELM1*/*2*, *PMD1*/*2* (reviewed in Arimura 2018). To evaluate the functions and contributions of these factors to mitochondrial fission, we generated double knock-out mutants for each factor using genome editing and analyzed mitochondrial morphology under the same conditions. Consequently, mitochondria in *drp3a/b* and *elm1/2* mutants were markedly elongated and reduced in number per cell, whereas mitochondria in *fis1a/1b* and *pmd1/2* mutants were similar to those of the wild type (WT). Furthermore, mitochondria in the *fis1a/1b/pmd1/2* quadruple mutant exhibited WT-like morphology, suggesting that FIS1 and PMD have little, or not always significant, contribution to mitochondrial fission in *Arabidopsis*.

The *Arabidopsis* dynamin-related proteins, DRP3A and DRP3B, are functionally redundant and play essential roles in mitochondrial fission (Arimura and Tsutsumi 2002; Arimura et al. 2004a; Logan et al. 2004; Fujimoto et al. 2009). Their yeast and mammalian orthologs, Dnm1/Drp1 (Bleazard et al. 1999; Smirnova et al. 2001; Figs. S1 and S2a), are cytosolic proteins forming polymeric ring-like structures at mitochondrial constriction sites, where they mediate division of the outer mitochondrial membrane (OMM) (Tábara et al. 2025). We previously reported that ELM1, a plant-specific OMM peripheral protein, recruits cytosolic DRP3A/3B to fission sites via protein-protein interaction (Arimura et al. 2008; Figs. S1 and S2b). Its paralog, ELM2, participates in mitochondrial fission with partial redundancy with ELM1 (Arimura et al. 2017). Moreover, there is an ELM1-independent/DRP3A-dependent mitochondrial division under cold temperature (Arimura et al. 2017). Double knock-out mutants of *DRP3A/3B* or *ELM1/2* exhibit severely elongated mitochondria and a marked reduction in mitochondrial number (Fujimoto et al. 2009; Arimura et al. 2017). In yeast, the tail-anchored OMM protein Fis1p recruits Dnm1p to mitochondrial fission sites via adaptors mitochondrial division protein 1 (Mdv1p) and its paralogue CRR_4_-associated factor 4 (Caf4p); consequently, loss of Fis1p results in fused mitochondria, resembling *dnm1* mutants phenotype (Tábara et al. 2025; Fig. S1). While human hFis1 acts as a receptor for Drp1 despite the absence of Mdv1p/Caf4p homologs, loss of hFis1 causes a milder mitochondrial fission defect than loss of another OMM receptor, mitochondrial fission factor (Mff). Thus, hFis1 plays a minor role in Drp1-mediated mitochondrial fission (Yoon et al. 2003; Otera et al. 2010; Losón et al. 2013; Fig. S1). In *Arabidopsis*, single gene mutants of the tail-anchored OMM proteins, *fis1A*, *fis1B*, *pmd1*, and *pmd2*, have been reported to exhibit fewer and enlarged mitochondria (Scott et al. 2006; Zhang and Hu 2008; Aung and Hu 2011; Figs. S1 and S2c, d). However, their mitochondrial morphology has not been compared with that of *drp3* mutants and *elm1* mutants under the same conditions. Given potential functional redundancy between the paralogs, their double knock-out analysis is necessary to elucidate their precise roles. To date, no double knock-out mutants of *FIS1* or *PMD* have been reported. In *Marchantia polymorpha*, which possesses a single copy of *DRP3*, *ELM1*, and *FIS1* per genome and lacks *PMD*, *drp3* mutant and *elm1* mutant showed severe mitochondrial elongation, whereas the mitochondrial morphology of *fis1* mutant resembled that of the WT (Nagaoka et al. 2017). Here, we generated *Arabidopsis* double knock-out mutants for all 4 factors using CRISPR/Cas9 system to directly compare mitochondrial phenotypes under the same conditions.

We designed pairs of guide RNAs (gRNAs) targeting the 5′ region of the 2 paralogous genes (Fig. 1a), and expressed them with Cas9 in *Arabidopsis* lines expressing mitochondrial-targeted GFP (Arimura et al. 2008). In the T_3_ generation, we established 4 double homozygous mutant lines without the CRISPR/Cas9 gene. Sequence analysis of genomic DNA confirmed 1 to 55 bp insertions or 10 bp deletions at the target sites, introducing premature termination codons within 1 to 49 amino acids downstream of the target sites (Fig. S3), which was further verified by RNA analysis. We grew these lines under the same conditions and observed mitochondria in cotyledon epidermal cells using confocal microscopy. Mitochondria labeled with GFP in the WT appeared as particle or rod-like structures (Fig. 1b). In contrast, mitochondria in *drp3a/b* and *elm1/2* mutants were markedly elongated and networked with some larger nodule-like mitochondria (∼2 *µ*m diameter). Conversely, mitochondria in *fis1a/1b* and *pmd1/2* mutants displayed WT-like morphology (Fig. 1b and c). Consistent trends were observed in independent experimental replicates using 3 individual plants per genotype (50 cells each; Fig. S7), confirming the difference between *drp3a/b*, *elm1/2* mutants and *fis1a/1b*, *pmd1/2* mutants is consistent. Similar results were observed in true leaf epidermal cells and root epidermal cells (Fig. S4a and b). To test for functional redundancy between FIS1 and PMD in mitochondrial fission, we generated a *fis1a/1b/pmd1/2* quadruple mutant by knocking out *FIS1A* and *FIS1B* in a *pmd1/2* mutant background (Fig. S5). Its mitochondria also exhibited WT-like morphology (Fig. 1d), suggesting little to no contribution of these factors to mitochondrial fission under these conditions.

**Figure 1:**
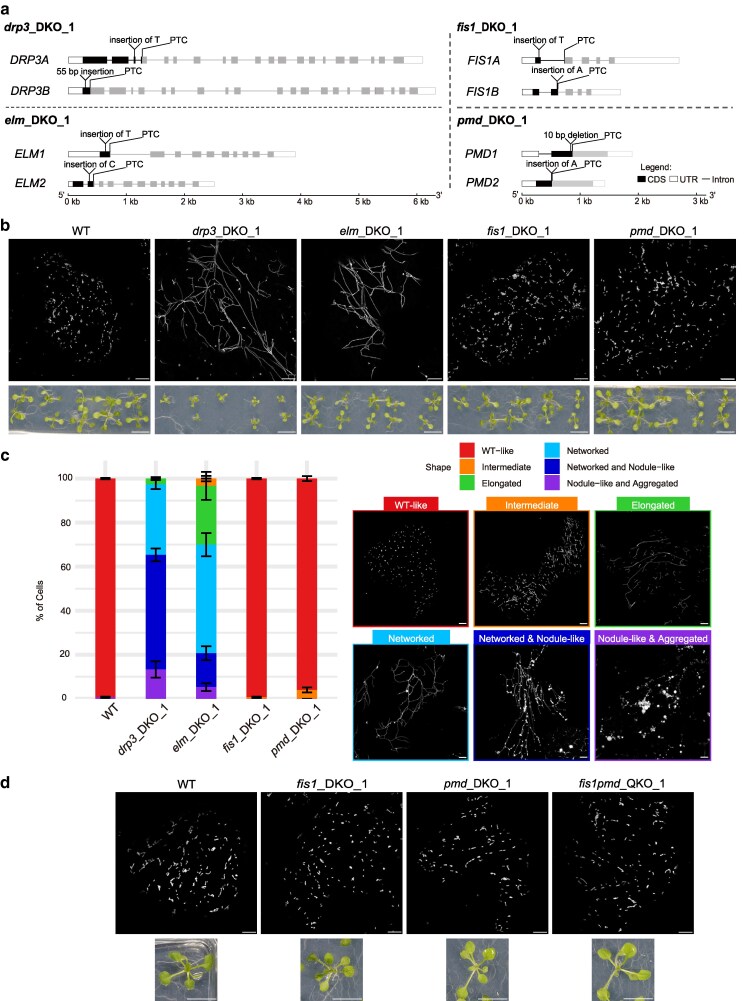
Characterization of Arabidopsis mitochondrial fission mutants. a) Gene diagrams indicating mutation sites. Filled boxes represent exons. Gray shading indicates predicted untranslated exons due to the premature termination codon. The mutation sites and gRNA sequences are shown in Fig. S3. b) Confocal microscopy images of mitochondria in cotyledon epidermal cells of *Arabidopsis* at 16 d after stratification (DAS) (top; bars = 10 *μ*m) and images of plants at 13 DAS (bottom; bars = 1 cm). c) Quantification of mitochondrial morphology of cotyledon epidermal cells at 15 DAS (*n* = 3 plants; error bars = Se). Representative classification images are shown in the right panel. d) Mitochondrial (at 18 DAS) and plant (at 14 DAS) phenotypes of double and quadruple (mitochondria; bars = 10 *µ*m, plants; bars = 1 cm). DKO, double knock-out; QKO, quadruple knock-out; PTC, premature termination codon.

*drp3a/b* mutants showed severe dwarfism and retardation of growth, consistent with Fujimoto et al. (2009), while *elm1/2* mutants showed weak dwarfism, consistent with Arimura et al. (2008). Thus, although mitochondria in *elm1/2* mutants elongated similarly to those of *drp3a/b* mutants, the impact of loss of ELM1/2 on plant growth was less severe than that of DRP3A/3B. *fis1a/1b* and *pmd1/2* plants were similar in size to the WT (Fig. 1b). This observation differs from the previous report describing growth defects in *fis1A* knock-out/*fis1B* knockdown mutants (Zhang and Hu 2008) but is consistent with the report where no clear growth inhibition was observed in *pmd1* knock-out/*pmd2* knockdown mutants (Aung and Hu 2011). Our results indicate that, under our experimental conditions, loss of FIS1 or PMD causes no obvious defects in plant size before bolting.

We generated double knock-out mutants to eliminate paralogous complementation. Our results suggested that loss of DRP3 or ELM1 reduces mitochondrial fission frequency, making mitochondrial fusion relatively dominant and causing elongation, whereas FIS1 and PMD may not be strongly involved in mitochondrial fission in cotyledons, true leaves, or roots epidermal cells under our experimental conditions. This aligns with findings in humans and liverworts, where Fis1 orthologs play a minor role in mitochondrial fission (Otera et al. 2010; Losón et al. 2013; Nagaoka et al. 2017). Similarly, PMD1/2 homologs show relatively low mutual sequence similarity, and they are present primarily in rosids (Fig. S2), suggesting they are unlikely to be broadly conserved mitochondrial fission factors. The *fis1a/1b/pmd1/2* quadruple mutant analysis further discounts the possibility of functional redundancy between these tail-anchored proteins. Although mammalian Drp1 utilizes Mff and MiD49/51 as alternative receptor proteins, clear homologs of these factors have not been identified in plants (Arimura 2018). While the possibility of other receptor factors (either proteins or lipids) cannot be entirely ruled out, the lack of distinct mitochondrial morphological differences between the *fis1a/1b/pmd1/2* quadruple mutant and the WT suggests that even their additive loss does not identify them as primary receptor proteins for fission in the tissues examined.

Despite similar mitochondrial elongation in *drp3a/3b* and *elm1/2* mutants, the plant phenotypes differed: only *drp3a/b* mutants exhibited severe dwarfism. This is likely attributable to functional differences between DRP3 and ELM1. First, DRP3A is also involved in peroxisomal fission, while ELM1 is not (Arimura et al. 2008; Fujimoto et al. 2009) Consistently, we observed enlarged and fewer peroxisomes in the *drp3a/3b* double mutant in this study (Fig. S6). These results suggest that the differences in plant growth between *drp3a/3b* and *elm1/2* may stem from differences in peroxisomal morphology. Notably, previous studies reported a reduced number of peroxisomes in the *fis1A* mutant (Zhang and Hu 2008) and enlarged peroxisomes in the *pmd1* mutant (Aung and Hu 2011). Another potential cause for the difference is that cold-induced transient mitochondrial fragmentation requires DRP3A but not ELM1/2 (Arimura et al. 2017). In our newly generated *drp3a/3b* and *elm1/2* mutants, both exhibited severely elongated mitochondrial morphology at room temperature; however, upon cold treatment, the mitochondrial morphology recovered to WT-like levels only in the *elm1/2* mutant (Fig. S7). The existence of such an ELM1-independent/DRP3-dependent mitochondrial fission mechanism likely contributes to the growth differences observed between these 2 mutants. Therefore, mitochondrial fission defects caused by loss of ELM1/2 appear to be milder than those caused by loss of DRP3A/3B, which may account for the difference in plant size. Future detailed research is expected to focus on functions of DRP3s, ELM1, and other factor(s) in plant mitochondrial fission as well as fusion.

## Supplementary Material

kiag531_Supplementary_Data

## Data Availability

The data and methods that support the findings of this study are available in the supplementary data of this article.
