# Supplementary material for: Comparative observation of mitochondrial morphology in Arabidopsis mutants of 4 reported fission factors
Source: Plant Physiol. 2026 Jul 30;201(3):kiag531. doi: 10.1093/plphys/kiag531 (PMC13421887; doi:10.1093/plphys/kiag531)
Supplement: kiag531_Supplementary_Data [file kiag531_supplementary_data.zip › Hashimoto-et-al-2026_SFig_revised2.pdf]

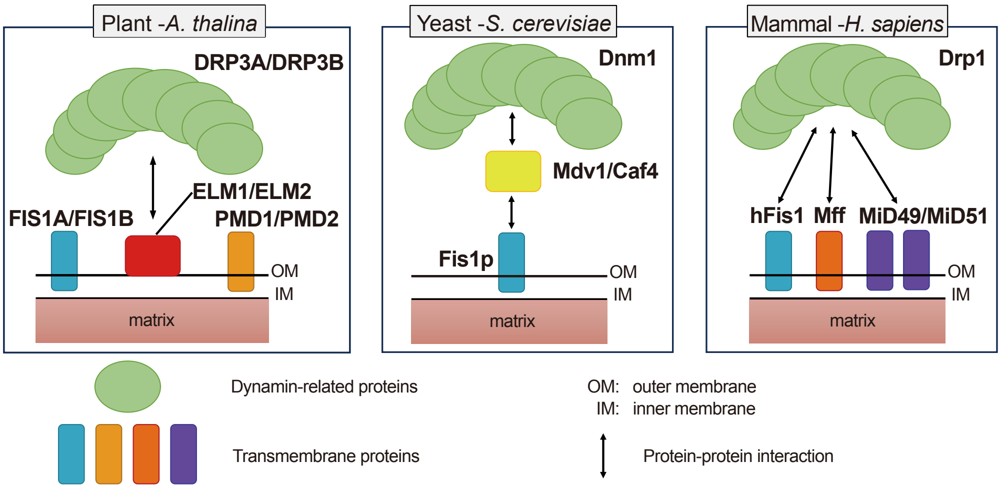

Supplementary Figure S1. The current models of mitochondrial fission in *Arabidopsis thaliana*, *Saccharomyces cerevisiae*, and *Homo sapiens*.

These figures are modified from Nagaoka *et al.*, 2017.

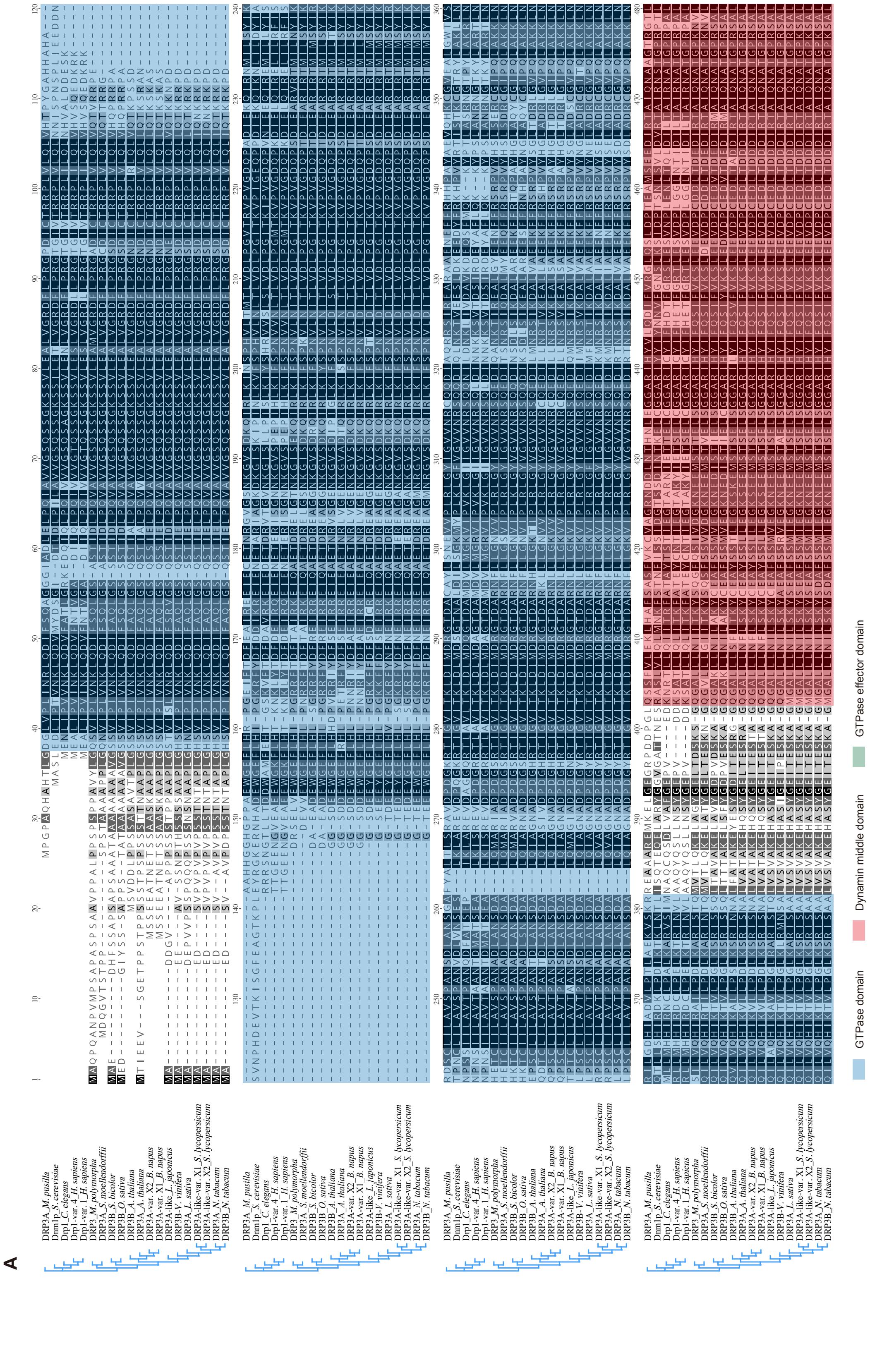
A
GTPase domain
Dynamin middle domain
GTPase effector domain

A (Continued)

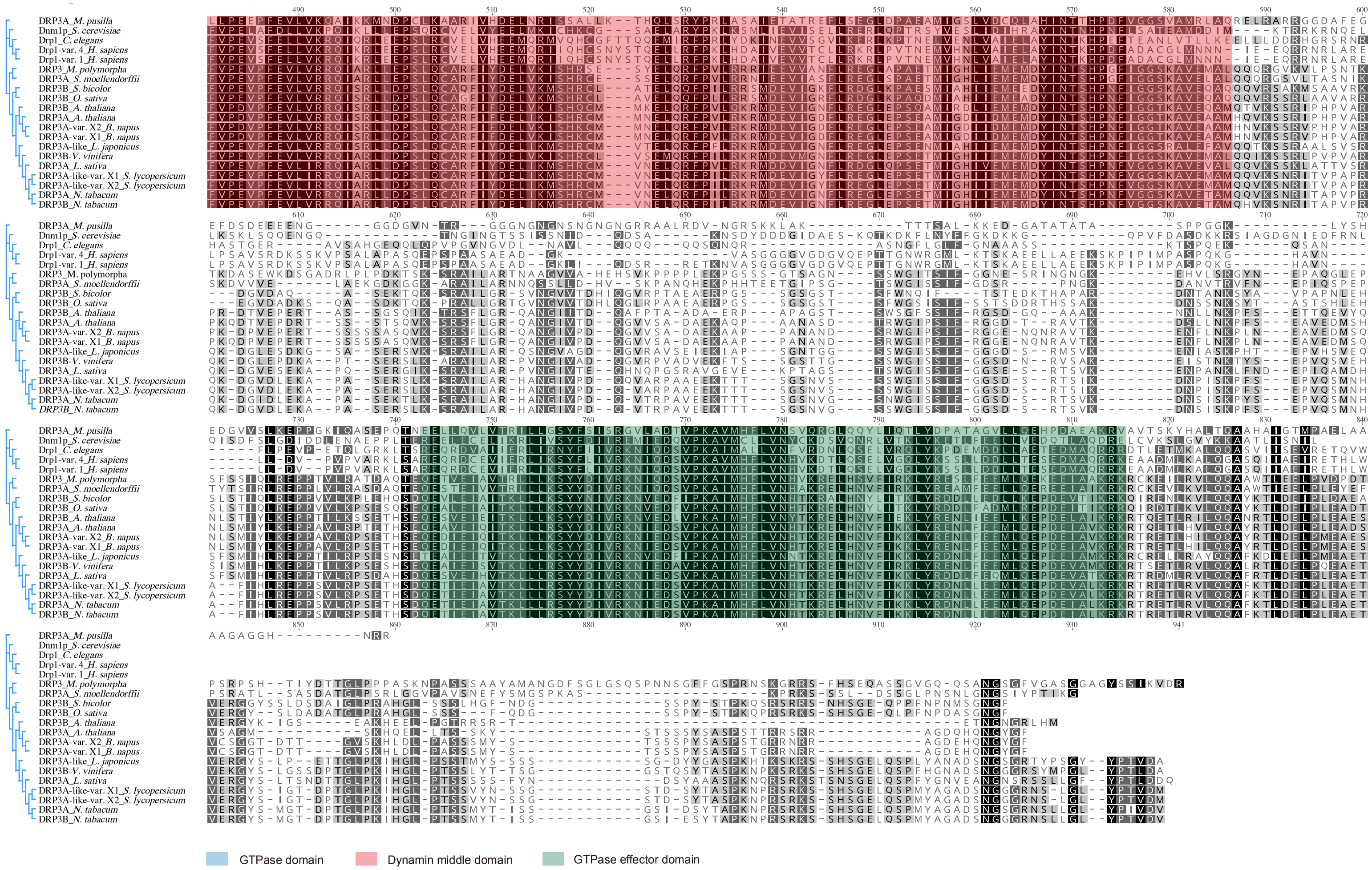
GTPase domain
Dynamin middle domain
GTPase effector domain

B

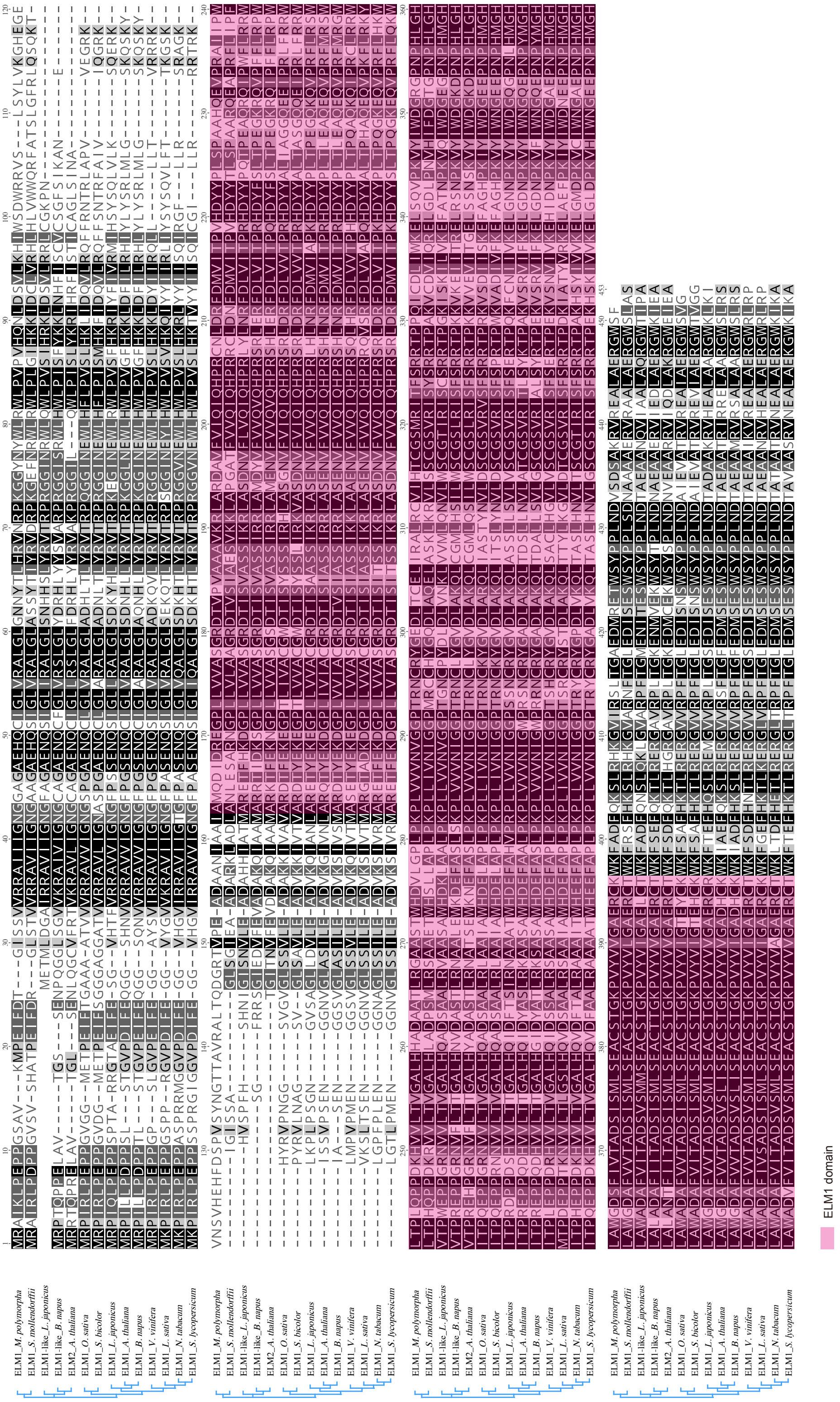
ELM1_M. polymorpha
ELM1_S. moellendorffii
ELM1-like_L. japonicus
ELM1-like_B. napus
ELM2_A. thaliana
ELM1_O. sativa
ELM1_S. bicolor
ELM1_L. japonicus
ELM1_A. thaliana
ELM1_B. napus
ELM1_V. vinifera
ELM1_L. sativa
ELM1_N. tabacum
ELM1_S. lycopersicum
ELM1 domain

C

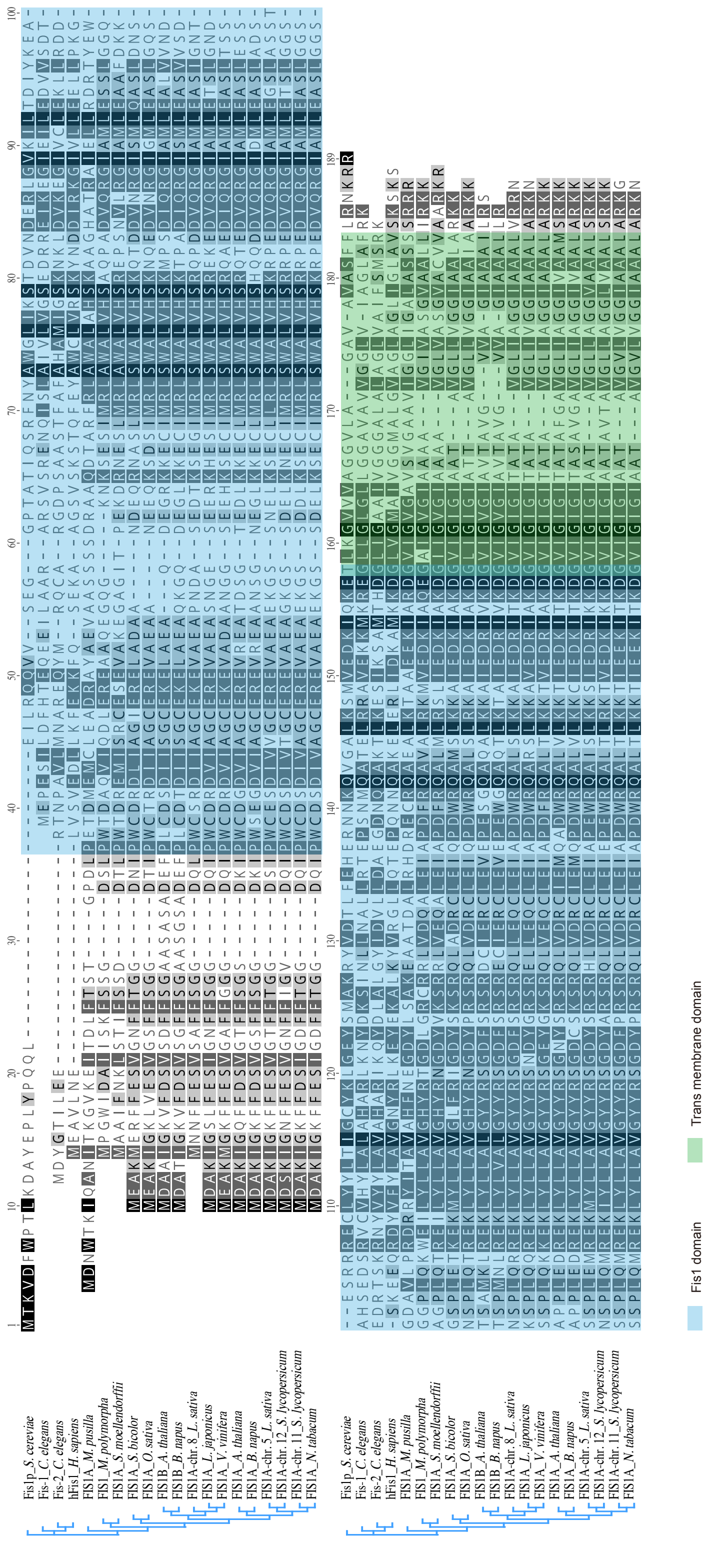

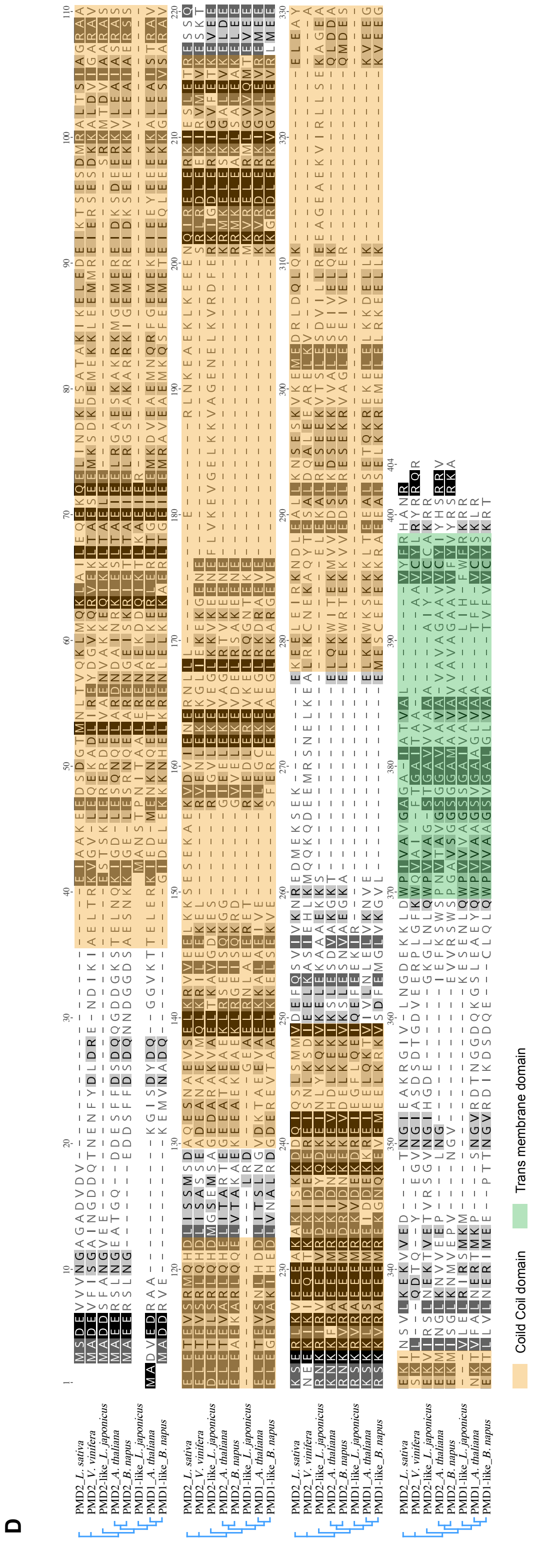
D
Coild Coil domain
Trans membrane domain

Supplemental Figure S2. Sequence alignment and phylogenetic analysis of fission factor homologs.

A) to **D)**, Amino acid sequence alignment of DRP3A/3B **(A)**, ELM1/2 **(B)**, FIS1A/1B **(C)**, and PMD1/2 **(D)** homologs. Representative homologous sequences of four fission factors were retrieved from *Saccharomyces cerevisiae*, *Homo sapiens*, *Caenorhabditis elegans*, *Marchantia polymorpha*, *Micromonas pusilla*, *Selaginella moellendorffii*, *Sorghum bicolor*, *Oryza sativa*, *Nicotiana tabacum*, *Brassica napus*, *Lotus japonicus*, *Vitis vinifera*, *Solanum lycopersicum*, and *Lactuca sativa*. Amino acid sequences of *Arabidopsis thaliana* DRP3A/B, ELM1/2, FIS1A/1B, and PMD1/2 were used as queries for BLASTP searches, selecting variants with the lowest E-values. If the nearest variants for the two homologs were identical, only one is shown; otherwise, both are displayed. Shaded boxes represent domains identified using InterPro (https://www.ebi.ac.uk/interpro, Blum et al., 2025) in *Arabidopsis* DRP3A, ELM1, FIS1A, and PMD1. Sequence alignments were generated using Clustal W, and the phylogenetic trees were constructed using the neighbor-joining method in Geneious Prime. All protein IDs are listed in Supplementary Table S1. var., variant or isoform; chr., chromosome.

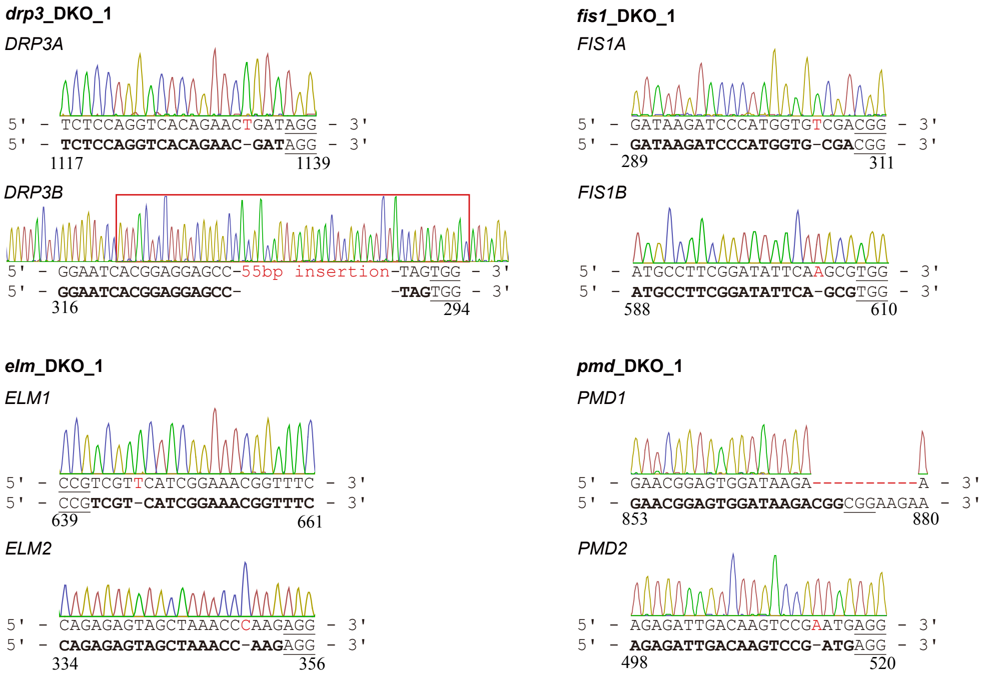

Supplementary Figure S3. Sequence verification of gRNA target sites in double knock-out mutants

Sanger chromatograms of genomic DNA from mutants are shown. Below the chromatogram, the mutant and wild-type sequences are shown as text at the top and bottom, respectively. Mutations are indicated in red, and the protospacer adjacent motifs are underlined. The guide RNAs target regions are bold. Numbers below the wild-type sequence indicate the nucleotide positions within the gene.

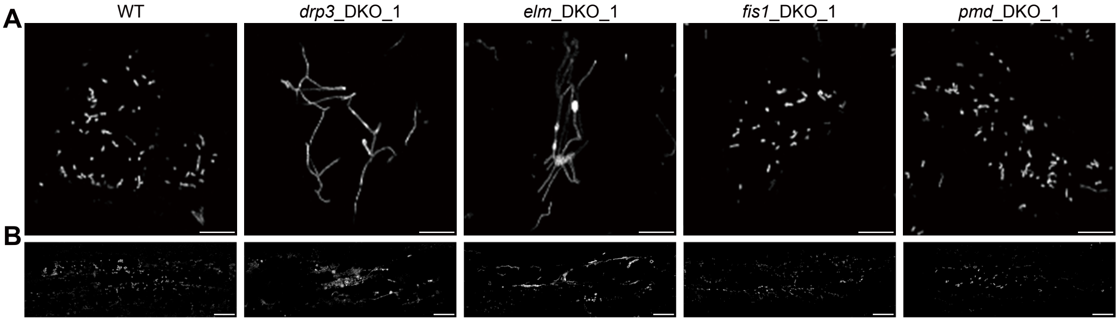

Supplementary Figure S4. Confocal microscopy images of mitochondria in true leaf epidermal cells and roots epidermal cells.

Confocal microscopy images of mitochondria in epidermal cells of *Arabidopsis* true leaves at 25 DAS **(A)** and roots at 11 DAS **(B)**. Scale bars = 10 μm.

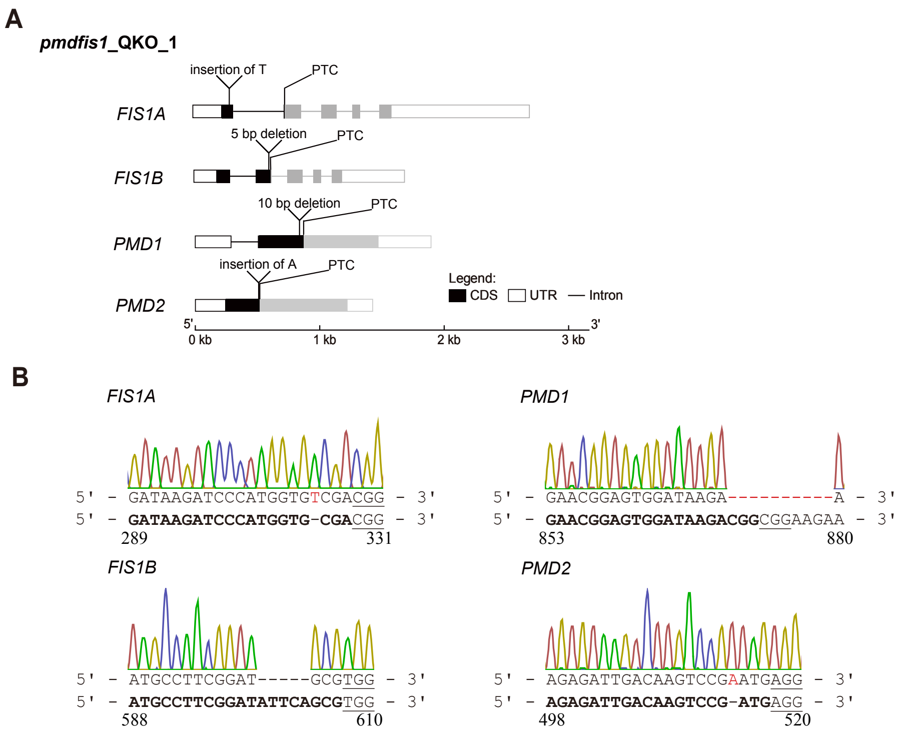

Supplementary Figure S5. Mutation types, positions and sequence verification in *fis1a/1b/pmd1/2* quadruple mutant.

A) Gene diagrams indicating the position of mutation. Filled boxes represent exons. Gray shading indicates predicted untranslated exons due to the premature termination codon. **B)** Genomic DNA sequence analysis of gRNA target sites in the T_3 generation. Below the Sanger chromatogram of mutants, the mutant and wild-type sequences are shown as text at the top and bottom, respectively. Mutations are indicated in red, and the protospacer adjacent motifs are underlined. The guide RNAs target regions are bold. Numbers below the wild-type sequence indicate the nucleotide positions within the gene. QKO, quadruple knock-out; PTC, premature termination codon.

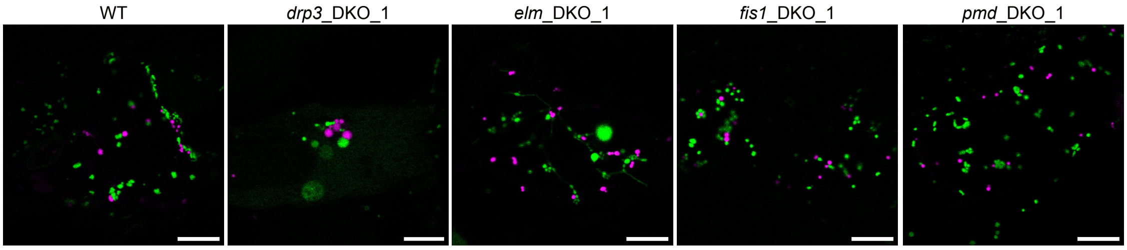

Supplementary Figure S6. Confocal microscopy images of peroxisomes and mitochondria in true leaf epidermal cells

Fluorescent protein construct visualizing peroxisomes by RFP (magenta) were transiently expressed in true leaf epidermal cells of Arabidopsis at 41 DAS. Mitochondria were simultaneously visualized using mt-GFP. Scale bars = 10 µm.

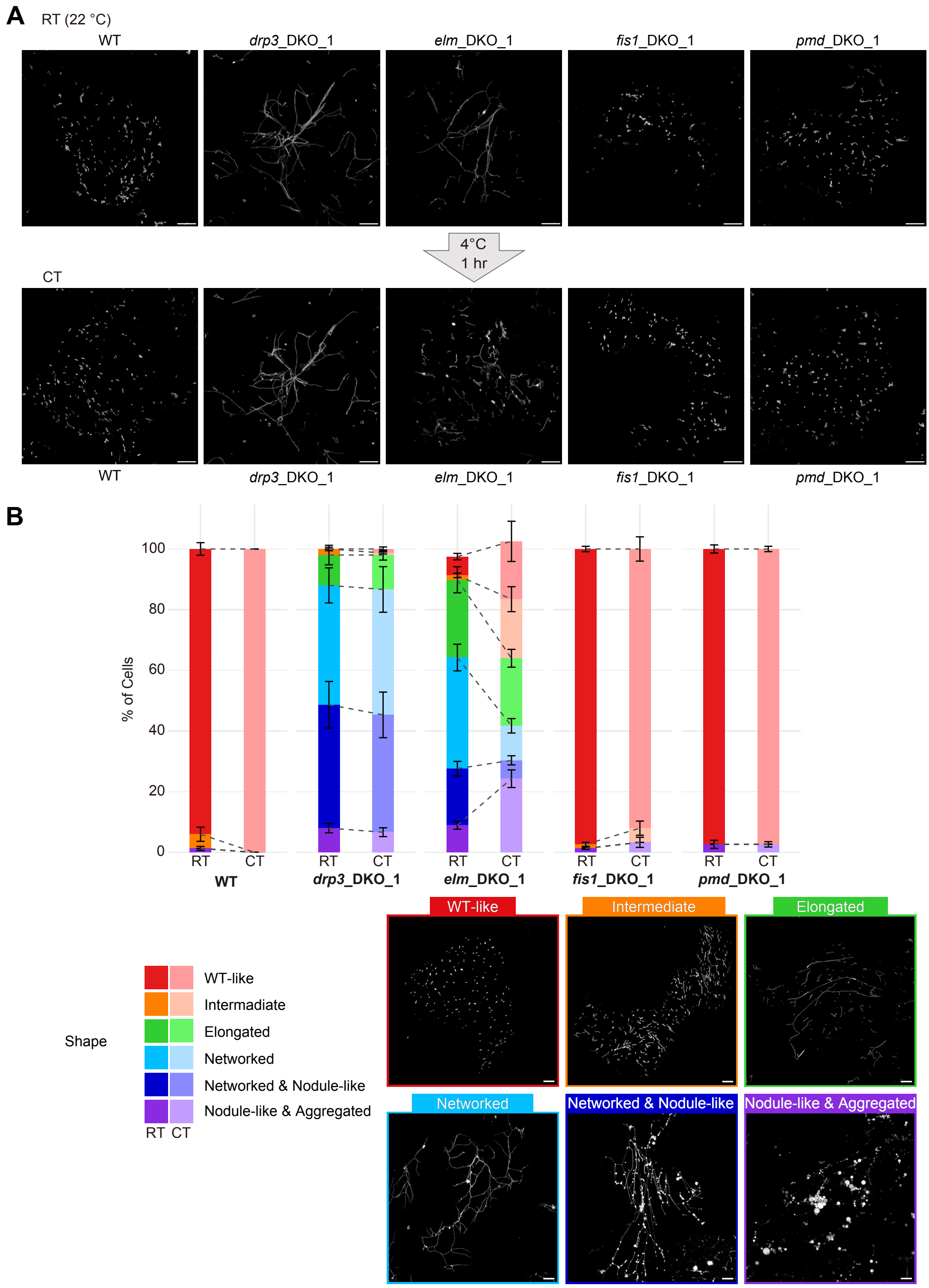

63

64 **Supplementary Figure S7. Mitochondrial response to cold treatment.**

A) Representative images of cotyledon epidermal cells at 19 DAS under 22°C (room temperature, RT) and after 1 hour incubation at 4°C (cold treatment, CT). Scale bars = 10 µm. **B)** Quantification of morphology of mitochondria of cotyledon epidermal cells at 15 DAS under RT and CT (white shading bars) conditions (n = 3; error bars = SE). Representative classification images are shown at the bottom right; these images are the same as those presented in Figure 1C. Scale bars = 10 µm. Asterisks indicate significant differences compared to RT ($P < 0.05$).
