# Supplementary material for: Comparative observation of mitochondrial morphology in Arabidopsis mutants of 4 reported fission factors
Source: Plant Physiol. 2026 Jul 30;201(3):kiag531. doi: 10.1093/plphys/kiag531 (PMC13421887; doi:10.1093/plphys/kiag531)
Supplement: kiag531_Supplementary_Data [file kiag531_supplementary_data.zip › Hashimoto-et-al-2026_Supplementary_Material_clean_260613.docx]

**Comparative observation of mitochondrial morphology in Arabidopsis mutants of four reported fission factors**

Masaru Hashimoto^1^, Yugo Ito^1^, Issei Nakazato^1^, Hideki Takanashi^1^, Shin-ichi Arimura^1*^

^1^ Graduate School of Agricultural and Life Sciences, The University of Tokyo, Bunkyo-ku, Tokyo 113-8657, Japan

*Author for correspondence: [arimura@g.ecc.u-tokyo.ac.jp](mailto:arimura@g.ecc.u-tokyo.ac.jp)

The author responsible for distribution of materials integral to the findings presented in this article in accordance with the policy described in the Instructions for Authors (https://academic.oup.com/plphys/pages/general-instructions) is Shin-ichi Arimura ([arimura@g.ecc.u-tokyo.ac.jp](mailto:arimura@g.ecc.u-tokyo.ac.jp)).

**Supplementary Materials and Methods**

**Plant Materials and Growth Conditions**

*Arabidopsis thaliana* transformant expressing mitochondrial-targeted GFP (Mt-GFP; Arimura et al., 2008) was used as a wild-type plant in this article. All *Arabidopsis* plants were grown at 22°C under long-day conditions (16h light, 8h dark) with a light intensity 50-150 µmol m^-2^ s^-1^. Seeds were sown on half-strength Murashige and Skoog (1/2MS) medium (pH 5.7) containing 2.3 g L^−1^ of MS Plant Salt Mixture (Wako), 0.5 g L^−1^ of MES, 10 g L^−1^ of sucrose, 1 mL L^−1^ of Gamborg’s Vitamin Solution (Sigma–Aldrich), 0.5 mL L^−1^ of Plant Preservative Mixture (Plant Cell Technology), and 6 g L^−1^ of agar. For root observations, plants were grown nearly vertically on the 1/2 MS medium containing 13 g L^−1^ of agarose. Seedlings at two- to three-week-old were transferred to Jiffy-7 (Jiffy Products International).

**Vector Construction**

CRISPR/Cas9 target sites were designed within the 1^st^ to 3^rd^ exons of *DRP3A, DRP3B, ELM1, ELM2, FIS1A, FIS1B, PMD1,* and *PMD2* using CHOPCHOP (Labun et al., 2019, <https://chopchop.cbu.uib.no>). All gRNA sequences are listed in Supplementary Table S1.

Vectors were constructed using Golden Gate Assembly (Vad-Nielsen et al., 2016) and In-Fusion cloning. Annealed gRNAs were cloned into the pEn-Chimera entry vector (Addgene ID #61432, Fauser et al., 2014) via Golden Gate Assembly. To generate double-targeting vectors (targeting *DRP3A*/*DRP3B, ELM1*/*ELM2, FIS1A*/*FIS1B*, and *PMD1*/*PMD2*), an additional gRNA cassette was assembled into the pEn-Chimera entry vector containing a *Cas9* expression cassette under the control of *RPS5A* promoter (Tsutsui & Higashiyama, 2017) using the In-Fusion^®^ HD Cloning Kit (Takara). Finally, the expression vectors containing two gRNAs and a Cas9 cassette were transferred into a Ti-plasmid containing the *Oleosin-GFP* selection marker (Shimada et al., 2010), via a Gateway LR reaction using LR Clonase II enzyme (Thermo Fisher Scientific).

**Generation of Transgenic Plants**

Plants were transformed via the floral dipping (Clough & Bent, 1998)with *Agrobacterium tumefaciens* strain C58C1 that harbored the CRISPR/Cas9 expression vector. Transgenic T_1_ seeds were selected based on seed GFP fluorescence (Ole-GFP). GFP-positive seeds were sown on the 1/2 MS medium with 125 mg L^−1^ of cefotaxime (Claforan). To obtain Cas9-free genome-edited mutants (null segregants) in the next generation, non-fluorescent (GFP-negative) T_2_ seeds were selected. Later, T_2_ and T_3_ seeds were sown on the 1/2 MS medium (see Plant Materials and Growth Conditions). Homozygosity of mutations was confirmed in the T_3_ generation for all double mutant lines, which are designated as *drp3*_DKO_1, *elm*_DKO_1, *fis1*_DKO_1, and *pmd*_DKO_1, respectively.

To generate quadruple knock-out (QKO) lines, the established *pmd*_DKO_1 line was transformed with the *FIS1A/1B*-targeting vector. Homozygous QKO lines (*fis1pmd*_QKO_1) were established in the T_4_ generation following similar selection procedures.

**Genotyping Analysis**

Total DNA was extracted from a young true leaf of the selected seedlings using a simplified isolation buffer (100 mM Tris-HCl, pH 9.5; 10 mM EDTA, pH 8.0). One rosette leaf from the seedlings was detached and placed into 50 µL of isolation buffer and incubated at 95°C for 15 minutes. Additionally, total RNA was isolated from a young true leaf of the selected seedlings with the ISOSPIN Plant RNA Kit (310-08171, Nippon Gene) and subsequently treated with RNase-free DNase I according to the manufacturer’s instructions. Target regions were amplified using specific primers (Supplementary Table S1).  Purified PCR products were subjected to Sanger sequencing (Eurofins Genomics and Fasmac) to detect mutations. The data were analyzed with Geneious Prime (v2026.0.2).

**Microscopic Observations and Image Analysis**

Image of plants at 13 d after stratification (DAS) were taken by SONY α6400. Mitochondria in epidermal cells of *Arabidopsis* cotyledons (plants at 16 DAS for Figure 1B and plants at 18 DAS for Figure 1D) and true leaves (plants at 25 DAS) were visualized using confocal laser scanning microscopy (STELLARIS 5, Leica). For quantitative analysis, 50 cells per replicate were classified into one of the six morphological categories under blind conditions (n = 3, plants at 19 DAS). Representative images are shown in Figure 1C. For cold treatment analysis, samples mounted on glass slides were incubated in 4°C in the dark, as described in our previous study (Arimura et al., 2017) prior to imaging and the same quantitative analysis was conducted. These images were processed with Adobe Photoshop 2026.

Statistical analyses were performed using RStudio (2023.09.1+494). Tukey’s multiple-comparison test was used to compare differences between lines within each morphological category. For the cold treatment analysis, Student's t-test was used to compare room temperature and cold conditions for each line within each category.

**Particle Bombardment**
Plasmids for visualizing mitochondria and peroxisomes of *Arabidopsis* leaves at 37 DAS were introduced via particle bombardment system (PDS-1000, Bio-Rad) according to the manufacturer’s instructions. To enhance delivery efficiency, a flow-guiding barrel (Thorpe et al., 2025) was employed. The bombardment parameters applied were as follows: bombardment pressure, 4.5 × 104 hPa; gold particles of 0.6 µm in diameter; a target distance of 15 cm; decompression vacuum, 950 hPa. After bombardment, plants were incubated for 1 d and observed using confocal laser scanning microscopy.

**Accession Numbers**

The AGI numbers for the gene in this study are *DRP3A*; AT4G33650, *DRP3B*; AT2G14120, *ELM1*; AT5G22350, *ELM2*; AT5G06180, *FIS1A*; AT3G57090, *FIS1B*; AT5G12390, *PMD1*; AT3G58840, *PMD2*; AT1G06530.

**References**

**Arimura, S. I., Fujimoto, M., Doniwa, Y., Kadoya, N., Nakazono, M., Sakamoto, W., & Tsutsumi, N. (2008). Arabidopsis elongated mitochondria1 is required for localization of dynamin-related protein3A to mitochondrial fission sites. *Plant Cell*, *20*(6), 1555–1566. https://doi.org/10.1105/tpc.108.058578**

**Arimura, S. I., Kurisu, R., Sugaya, H., Kadoya, N., & Tsutsumi, N. (2017). Cold treatment induces transient mitochondrial fragmentation in Arabidopsis thaliana in a way that requires DRP3A but not ELM1 or an ELM1-like homologue, ELM2. *International Journal of Molecular Sciences*, *18*(10). https://doi.org/10.3390/ijms18102161**

**Blum, M., Andreeva, A., Florentino, L. C., Chuguransky, S. R., Grego, T., Hobbs, E., Pinto, B. L., Orr, A., Paysan-Lafosse, T., Ponamareva, I., Salazar, G. A., Bordin, N., Bork, P., Bridge, A., Colwell, L., Gough, J., Haft, D. H., Letunic, I., Llinares-López, F., … Bateman, A. (2025). InterPro: the protein sequence classification resource in 2025. *Nucleic Acids Research*, *53*(D1), D444–D456. https://doi.org/10.1093/nar/gkae1082**

**Clough, S. J., & Bent, A. F. (1998). Floral dip: a simplified method for Agrobacterium‐mediated transformation of Arabidopsis thaliana. *The Plant Journal*, *16*(6), 735–743.**

**Fauser, F., Schiml, S., & Puchta, H. (2014). Both CRISPR/C as‐based nucleases and nickases can be used efficiently for genome engineering in A rabidopsis thaliana. *The Plant Journal*, *79*(2), 348–359.**

**Labun, K., Montague, T. G., Krause, M., Torres Cleuren, Y. N., Tjeldnes, H., & Valen, E. (2019). CHOPCHOP v3: expanding the CRISPR web toolbox beyond genome editing. *Nucleic Acids Research*, *47*(W1), W171–W174. https://doi.org/10.1093/nar/gkz365**

**Shimada, T. L., Shimada, T., & Hara-Nishimura, I. (2010). A rapid and non-destructive screenable marker, FAST, for identifying transformed seeds of Arabidopsis thaliana. *The Plant Journal*, *61*(3), 519–528. https://doi.org/https://doi.org/10.1111/j.1365-313X.2009.04060.x**

**Thorpe, C., Luo, W., Ji, Q., Eggenberger, A. L., Chicowski, A. S., Xu, W., Sandhu, R., Lee, K., Whitham, S. A., Qi, Y., Wang, K., & Jiang, S. (2025). Enhancing biolistic plant transformation and genome editing with a flow guiding barrel. *Nature Communications*, *16*(1), 5624. https://doi.org/10.1038/s41467-025-60761-x**

**Tsutsui, H., & Higashiyama, T. (2017). pKAMA-ITACHI vectors for highly efficient CRISPR/Cas9-mediated gene knockout in Arabidopsis thaliana. *Plant and Cell Physiology*, *58*(1), 46–56.**

**Vad-Nielsen, J., Lin, L., Bolund, L., Nielsen, A. L., & Luo, Y. (2016). Golden Gate Assembly of CRISPR gRNA expression array for simultaneously targeting multiple genes. *Cellular and Molecular Life Sciences*, *73*(22), 4315–4325.**

**Supplementary Table S1**

DRP3A/3B, ELM1/2, FIS1A/1B, and PMD1/2 homologs collected from NCBI database and Phytozome (for liverwort) and used to construct the crustal arraignment (Supplementary Figure S2).

| Accession number | Protein name | Nearest homolog in *Arabidopsis* | Scientific name of each species |
| --- | --- | --- | --- |
| NP_013100.1 | dynamin-related GTPase DNM1 | DRP3A | *Saccharomyces cerevisiae* |
| NP_741403.2 | Dynamin-1-like protein drp-1 | DRP3A | *Caenorhabditis elegans* |
| [NP_036192.2](https://www.ncbi.nlm.nih.gov/protein/NP_036192.2?report=genbank&log$=prottop&blast_rank=2003&RID=PPK4V2YF016) | dynamin-1-like protein isoform 1 | DRP3A | *Homo sapiens* |
| NP_001265392.1 | dynamin-1-like protein isoform 4 | DRP3B | *Homo sapiens* |
| [XP_003063165.1](https://www.ncbi.nlm.nih.gov/protein/XP_003063165.1?report=genbank&log$=prottop&blast_rank=1687&RID=PPK4V2YF016) | uncharacterized protein MICPUCDRAFT_36259 | DRP3A | *Micromonas pusilla* CCMP1545 |
| Mapoly0069s0084.1.p | Mp*DRP3* | DRP3A | *Marchantia polymorpha* |
| XP_024529684.1 | dynamin-related protein 3A isoform X1 | DRP3A | *Selaginella moellendorffii* |
| [XP_015614254.1](https://www.ncbi.nlm.nih.gov/protein/XP_015614254.1?report=genbank&log$=prottop&blast_rank=488&RID=PPK4V2YF016) | dynamin-related protein 3B isoform X2 | DRP3A | *Oryza sativa Japonica Group* |
| XP_002458996.1 | dynamin-related protein 3B | DRP3A | *Sorghum bicolor* |
| XP_002269774.2 | dynamin-related protein 3B isoform X1 | DRP3A | *Vitis vinifera* |
| XP_057429210.1 | dynamin-related protein 3A-like | DRP3A | *Lotus japonicus* |
| [XP_013735194.1](https://www.ncbi.nlm.nih.gov/protein/XP_013735194.1?report=genbank&log$=prottop&blast_rank=12&RID=PPK4V2YF016) | dynamin-related protein 3A isoform X1 | DRP3A | *Brassica napus* |
| [XP_013735195.1](https://www.ncbi.nlm.nih.gov/protein/XP_013735195.1?report=genbank&log$=prottop&blast_rank=15&RID=PPNBURJF016) | dynamin-related protein 3A isoform X2 | DRP3B | *Brassica napus* |
| NP_001190906.1 | dynamin-related protein 3A |  | *Arabidopsis thaliana* |
| NP_565363.2 | dynamin related protein |  | *Arabidopsis thaliana* |
| [XP_075110856.1](https://www.ncbi.nlm.nih.gov/protein/XP_075110856.1?report=genbank&log$=prottop&blast_rank=113&RID=PPK4V2YF016) | dynamin-related protein 3A | DRP3A | *Nicotiana tabacum* |
| [XP_016471132.2](https://www.ncbi.nlm.nih.gov/protein/XP_016471132.2?report=genbank&log$=prottop&blast_rank=83&RID=PPNBURJF016) | dynamin-related protein 3B isoform X1 | DRP3B | *Nicotiana tabacum* |
| [XP_004250727.2](https://www.ncbi.nlm.nih.gov/protein/XP_004250727.2?report=genbank&log$=prottop&blast_rank=138&RID=PPN6ZH6H016) | dynamin-related protein 3A-like isoform X2 | DRP3A | *Solanum lycopersicum* |
| [XP_010313067.2](https://www.ncbi.nlm.nih.gov/protein/XP_010313067.2?report=genbank&log$=prottop&blast_rank=123&RID=PPNBURJF016) | dynamin-related protein 3A-like isoform X1 | DRP3B | *Solanum lycopersicum* |
| XP_023764759.1 | dynamin-related protein 3A | DRP3A | *Lactuca sativa* |
| Mapoly0038s0050.1.p | Mp*ELM1* | ELM1 | *Marchantia polymorpha* |
| XP_024543424.1 | mitochondrial fission protein ELM1 | ELM1 | *Selaginella moellendorffii* |
| [XP_015627638.1](https://www.ncbi.nlm.nih.gov/protein/XP_015627638.1?report=genbank&log$=prottop&blast_rank=299&RID=PPNCJ3CK014) | mitochondrial fission protein ELM1 | ELM1 | *Oryza sativa* |
| XP_002452214.1 | mitochondrial fission protein ELM1 | ELM1 | *Sorghum bicolor* |
| XP_002273670.1 | mitochondrial fission protein ELM1 | ELM1 | *Vitis vinifera* |
| XP_057455952.1 | mitochondrial fission protein ELM1 | ELM1 | *Lotus japonicus* |
| [XP_013667199.2](https://www.ncbi.nlm.nih.gov/protein/XP_013667199.2?report=genbank&log$=prottop&blast_rank=8&RID=PPNCJ3CK014) | mitochondrial fission protein ELM1 | ELM1 | *Brassica napus* |
| [XP_048623196.1](https://www.ncbi.nlm.nih.gov/protein/XP_048623196.1?report=genbank&log$=prottop&blast_rank=9&RID=PPNCYPMN016) | mitochondrial fission protein ELM1-like | ELM2 | *Brassica napus* |
| NP_568417.1 | fission ELM1-like protein (DUF1022) |  | *Arabidopsis thaliana* |
| NP_568162.1 | fission ELM1-like protein (DUF1022) |  | *Arabidopsis thaliana* |
| [XP_016492066.1](https://www.ncbi.nlm.nih.gov/protein/XP_016492066.1?report=genbank&log$=prottop&blast_rank=176&RID=PPNCJ3CK014) | mitochondrial fission protein ELM1 | ELM1 | *Nicotiana tabacum* |
| XP_004251072.1 | mitochondrial fission protein ELM1 | ELM1 | *Solanum lycopersicum* |
| XP_023768961.1 | mitochondrial fission protein ELM1 | ELM1 | *Lactuca sativa* |
| NP_012199.3 | Fis1p | FIS1A | *Saccharomyces cerevisiae* |
| NP_001024560.1 | FIS1-related protein fis-2 | FIS1A | *Caenorhabditis elegans* |
| NP_495381.1 | FIS1-related protein fis-1 | FIS1B | *Caenorhabditis elegans* |
| NP_057152.2 | mitochondrial fission 1 protein | FIS1A | *Homo sapiens* |
| XP_003058159.1 | uncharacterized protein MICPUCDRAFT_57361 | FIS1A | *Micromonas pusilla* CCMP1545 |
| Mapoly0147s0019.1.p | Mp*FIS1* | FIS1A | *Marchantia polymorpha* |
| XP_024540050.1 | mitochondrial fission 1 protein A | FIS1A | *Selaginella moellendorffii* |
| [XP_015640604.1](https://www.ncbi.nlm.nih.gov/protein/XP_015640604.1?report=genbank&log$=prottop&blast_rank=258&RID=PPNDTYU3016) | mitochondrial fission 1 protein A | FIS1A | *Oryza sativa Japonica Group* |
| XP_002441020.1 | mitochondrial fission 1 protein A | FIS1A | *Sorghum bicolor* |
| XP_003632733.1 | mitochondrial fission 1 protein A | FIS1A | *Vitis vinifera* |
| XP_057437976.1 | mitochondrial fission 1 protein A | FIS1A | *Lotus japonicus* |
| [XP_048597169.1](https://www.ncbi.nlm.nih.gov/protein/XP_048597169.1?report=genbank&log$=prottop&blast_rank=14&RID=PPNDTYU3016) | mitochondrial fission 1 protein A | FIS1A | *Brassica napus* |
| [XP_013676085.1](https://www.ncbi.nlm.nih.gov/protein/XP_013676085.1?report=genbank&log$=prottop&blast_rank=7&RID=PPZH9RAW016) | mitochondrial fission 1 protein B | FIS1B | *Brassica napus* |
| NP_001327069.1 | Tetratricopeptide repeat (TPR)-like superfamily protein |  | *Arabidopsis thaliana* |
| NP_568272.1 | Tetratricopeptide repeat (TPR)-like superfamily protein |  | *Arabidopsis thaliana* |
| [XP_016435272.1](https://www.ncbi.nlm.nih.gov/protein/XP_016435272.1?report=genbank&log$=prottop&blast_rank=354&RID=PPNDTYU3016) | mitochondrial fission 1 protein A-like | FIS1A | *Nicotiana tabacum* |
| [XP_004250907.2](https://www.ncbi.nlm.nih.gov/protein/XP_004250907.2?report=genbank&log$=prottop&blast_rank=25&RID=PPNDTYU3016) | mitochondrial fission 1 protein A | FIS1A | *Solanum lycopersicum* |
| [XP_004252888.1](https://www.ncbi.nlm.nih.gov/protein/XP_004252888.1?report=genbank&log$=prottop&blast_rank=22&RID=PPZH9RAW016) | mitochondrial fission 1 protein A | FIS1B | *Solanum lycopersicum* |
| [XP_023748993.1](https://www.ncbi.nlm.nih.gov/protein/XP_023748993.1?report=genbank&log$=prottop&blast_rank=65&RID=PPNDTYU3016) | mitochondrial fission 1 protein A | FIS1A | *Lactuca sativa* |
| [XP_023741161.1](https://www.ncbi.nlm.nih.gov/protein/XP_023741161.1?report=genbank&log$=prottop&blast_rank=55&RID=PPZH9RAW016) | tetratricopeptide repeat (TPR)-like superfamily protein | FIS1B | *Lactuca sativa* |
| XP_002283816.1 | peroxisomal and mitochondrial division factor 2 | PMD1 | *Vitis vinifera* |
| [XP_057430113.1](https://www.ncbi.nlm.nih.gov/protein/XP_057430113.1?report=genbank&log$=prottop&blast_rank=230&RID=PS8D03PW014) | peroxisomal and mitochondrial division factor 1-like | PMD1 | *Lotus japonicus* |
| [XP_057441309.1](https://www.ncbi.nlm.nih.gov/protein/XP_057441309.1?report=genbank&log$=prottop&blast_rank=144&RID=PS8DC7BF014) | peroxisomal and mitochondrial division factor 2-like | PMD2 | *Lotus japonicus* |
| [XP_013673077.2](https://www.ncbi.nlm.nih.gov/protein/XP_013673077.2?report=genbank&log$=prottop&blast_rank=11&RID=PS8D03PW014) | peroxisomal and mitochondrial division factor 1-like | PMD1 | *Brassica napus* |
| [XP_013723239.2](https://www.ncbi.nlm.nih.gov/protein/XP_013723239.2?report=genbank&log$=prottop&blast_rank=9&RID=PS8DC7BF014) | peroxisomal and mitochondrial division factor 2 | PMD2 | *Brassica napus* |
| [NP_001078312.1](https://www.ncbi.nlm.nih.gov/protein/NP_001078312.1?report=genbank&log$=prottop&blast_rank=1&RID=PS8D03PW014) | Tropomyosin-like protein |  | *Arabidopsis thaliana* |
| NP_172140.1 | Tropomyosin-like protein |  | *Arabidopsis thaliana* |
| XP_023735087.1 | peroxisomal and mitochondrial division factor 2 | PMD1 | *Lactuca sativa* |

**Supplementary Table S2**

List of primer pairs used in this study.

| **Primer** | **Sequence 5' to 3'** | **Purpose** |
| --- | --- | --- |
| DRP3A_gRNA_Fw | attgGGAATCACGGAGGAGCCTAG | sgRNA of DRP3A |
| DRP3A_gRNA_Rv | aaacCTAGGCTCCTCCGTGATTCC |  |
| DRP3B_gRNA_Fw | attgTCTCCAGGTCACAGAACGAT | sgRNA of DRP3B |
| DRP3B_gRNA_Rv | aaacATCGTTCTGTGACCTGGAGA |  |
| ELM1_gRNA_Fw | attgGAAACCGTTTCCGATGACGA | sgRNA of ELM1 |
| ELM1_gRNA_Rv | aaacTCGTCATCGGAAACGGTTTC |  |
| ELM2_gRNA_Fw | attgCAGAGAGTAGCTAAACCAAG | sgRNA of ELM2 |
| ELM2_gRNA_Rv | aaacCTTGGTTTAGCTACTCTCTG |  |
| FIS1A_gRNA_Fw | attgGATAAGATCCCATGGTGCGA | sgRNA of FIS1A |
| FIS1A_gRNA_Rv | aaacTCGCACCATGGGATCTTATC |  |
| FIS1B_gRNA_Fw | attgATGCCTTCGGATATTCAGCG | sgRNA of FIS1B |
| FIS1B_gRNA_Rv | aaacCGCTGAATATCCGAAGGCAT |  |
| PMD1_gRNA_Fw | attgGAACGGAGTGGATAAGACGG | sgRNA of PMD1 |
| PMD1_gRNA_Rv | aaacCCGTCTTATCCACTCCGTTC |  |
| PMD2_gRNA_Fw | attgAGAGATTGACAAGTCCGATG | sgRNA of PMD2 |
| PMD2_gRNA_Rv | aaacCATCGGACTTGTCAATCTCT |  |
| infusion_Fw | GTGCTTTTTTTCTAGGCTCTTTTTTTCTTCTTCTT | For in-fusion reaction |
| infusion_Rv | GAAAGCTGGGTCTAGAAAAA |  |
| DRP3A_Fw | GATCCTCCTCTCTTCGATAG | amplification of DRP3A (gDNA) target site |
| DRP3A_Rv | GCAACCACAGGTTTACCTCC |  |
| DRP3B_Fw | AGGGTTTACCTCTTGACTAC | amplification of DRP3B (gDNA) target site |
| DRP3B_Rv | GATTTACTTTAATCGGCGCG |  |
| ELM1_Fw | ATTCGTGATGATGGAAAGGC | amplification of ELM1 (gDNA) target site |
| ELM1_Rv | CTCTTTCTGTGACCTTTCGC |  |
| ELM2_Fw | TTAATTCGTTCCGATTCCCG | amplification of ELM2 (gDNA) target site |
| ELM2_Rv | TACCAGACGCCACGACTAAC |  |
| FIS1A_Fw | TGGTGATCAATGTGGGTAAG | amplification of FIS1A (gDNA) target site |
| FIS1A_Rv | GCTCCTTGAGTAATTCCCAC |  |
| FIS1B_Fw | CTTGAAAAGTCACCGCTTCG | amplification of FIS1B (gDNA) target site |
| FIS1B_Rv | GCCGTCAAATTTCTGGACTG |  |
| PMD1_Fw | CGCCATTTGTTGTCACTCAC | amplification of PMD1 (gDNA) target site |
| PMD1_Rv | CAACTCCAGCTCCTTCTCTC |  |
| PMD2_Fw | GTTAACATTGCTCTGTGCCC | amplification of PMD2 (gDNA) target site |
| PMD2_Rv | CTCCTTCACCTCCAAAGCAC |  |
| DRP3A_RTPCR_Fw | ATGACTATTGAAGAAGTTTCCGGTG | amplification of DRP3A (cDNA) target site |
| DRP3A_RTPCR_Rv | GAATCCGTATCCATTTTGGTGTTG |  |
| DRP3B_RTPCR_Fw | TTACATATGAAGCCGTCCGT | amplification of DRP3B (cDNA) target site |
| DRP3B_RTPCR_Rv | ATGTCCGTCGACGATCTCCC |  |
| ELM1_RTPCR_Fw | ATGAGGCCAATCCTATTGC | amplification of ELM1 (cDNA) target site |
| ELM1_RTPCR_Rv | TCAAGACCGTAAACTCCATCC |  |
| ELM2_RTPCR_Fw | ATGCGACGAACACAGCCA | amplification of ELM2 (cDNA) target site |
| ELM2_RTPCR_Rv | TCAAAGGTCGAACAGCTCC |  |
| FIS1A_RTPCR_Fw | ATGGATGCTAAGATCGGACA | amplification of FIS1A (cDNA) target site |
| FIS1A_RTPCR_Rv | TCATTTCTTGCGAGACATCG |  |
| FIS1B_RTPCR_Fw | ATGGACGCGGCGATAGG | amplification of FIS1B (cDNA) target site |
| FIS1B_RTPCR_Rv | TTAGCTGCGTAATATGGCTGC |  |
| PMD1_RTPCR_Fw | ATGGCGGATGTTGAAGAT | amplification of PMD1 (cDNA) target site |
| PMD1_RTPCR_Rv | TCACCTTAGCTTAGAATAGCAGACG |  |
| PMD2_RTPCR_Fw | ATGGCGGAAGAGAGGAGCTT | amplification of PMD2 (cDNA) target site |
| PMD2_RTPCR_Rv | TCAAACCCTCCTCGAGTGGT |  |
| DRP3A1_Fw | GGAGAGAACAAAGGTGTAGC | For sanger sequence of gDNA |
| DRP3B1_Fw | GTGATACCAGGAAGATCCAC |  |
| ELM1_Rv | CTCTTTCTGTGACCTTTCGC |  |
| ELM2_Rv | TACCAGACGCCACGACTAAC |  |
| FIS1A_Fw | TGGTGATCAATGTGGGTAAG |  |
| FIS1B_Fw | CTTGAAAAGTCACCGCTTCG |  |
| PMD1_Fw | CGCCATTTGTTGTCACTCAC |  |
| PMD2_Rv | CTCCTTCACCTCCAAAGCAC |  |
| DRP3A1_Fw | GGAGAGAACAAAGGTGTAGC | For sanger sequence of cDNA |
| DRP3B2_Rv | GCGAATCTCAGAGAAATCGT |  |
| ELM1_RTPCR_Fw | ATGAGGCCAATCCTATTGC |  |
| ELM2_RTPCR_Fw | ATGCGACGAACACAGCCA |  |
| FIS1A_RTPCR_Rv | TCATTTCTTGCGAGACATCG |  |
| FIS1B_RTPCR_Fw | ATGGACGCGGCGATAGG |  |
| PMD1_RTPCR_Fw | ATGGCGGATGTTGAAGAT |  |
| PMD2_RTPCR_Fw | ATGGCGGAAGAGAGGAGCTT |  |

**Supplementary Figure**

**Supplementary Figure S1. The current models of mitochondrial fission in *Arabidopsis* *thaliana*, *Saccharomyces* *cerevisiae*, and *Homo sapiens*.**

These figures are modified from Nagaoka *et al*., 2017.

**Supplementary Figure S2. Sequence alignment and phylogenetic analysis of fission factor homologs.**

**A)** to **D)**, Amino acid sequence alignment of DRP3A/3B **(A)**, ELM1/2 **(B)**, FIS1A/1B **(C)**, and PMD1/2 **(D)** homologs. Representative homologous sequences of four fission factors were retrieved from *Saccharomyces cerevisiae*, *Homo sapiens*, *Caenorhabditis elegans*, *Marchantia polymorpha*, *Micromonas pusilla*, *Selaginella moellendorffii*, *Sorghum bicolor*, *Oryza sativa*, *Nicotiana tabacum*, *Brassica napus*, *Lotus japonicus*, *Vitis vinifera*, *Solanum lycopersicum*, and *Lactuca sativa*. Amino acid sequences of *Arabidopsis thaliana* DRP3A/B, ELM1/2, FIS1A/1B, and PMD1/2 were used as queries for BLASTP searches, selecting variants with the lowest E-values. If the nearest variants for the two homologs were identical, only one is shown; otherwise, both are displayed. Shaded boxes represent domains identified using InterPro (<https://www.ebi.ac.uk/interpro>, Blum et al., 2025) in *Arabidopsis* DRP3A, ELM1, FIS1A, and PMD1. Sequence alignments were generated using Clustal W, and the phylogenetic trees were constructed using the neighbor-joining method in Geneious Prime. All protein IDs are listed in Supplementary Table S1. var., variant or isoform; chr., chromosome.

**Supplementary Figure S3. Sequence verification of gRNA target sites in double knock-out mutants**

Sanger chromatograms of genomic DNA from mutants are shown. Below the chromatogram, the mutant and wild-type sequences are shown as text at the top and bottom, respectively. Mutations are indicated in red, and the protospacer adjacent motifs are underlined. The guide RNAs target regions are bold. Numbers below the wild-type sequence indicate the nucleotide positions within the gene.

**Supplementary Figure S4. Confocal microscopy images of mitochondria in true leaf epidermal cells and roots epidermal cells.**

Confocal microscopy images of mitochondria in epidermal cells of *Arabidopsis* true leaves at 25 DAS **(A)** and roots at 11 DAS **(B)**. Scale bars = 10 μm.

**Supplementary Figure S5. Mutation types, positions and sequence verification in *fis1a/1b/pmd1/2* quadruple mutant.**

**A)** Gene diagrams indicating the position of mutation. Filled boxes represent exons. Gray shading indicates predicted premature proteins. **B)** Genomic DNA sequence analysis of gRNA target sites in the T_4_ generation. Below the Sanger chromatogram of mutants, the mutant and wild-type sequences are shown as text at the top and bottom, respectively. Mutations are indicated in red, and the protospacer adjacent motifs are underlined. The guide RNAs target regions are bold. Numbers below the wild-type sequence indicate the nucleotide positions within the gene. QKO, quadruple knock-out; PTC, premature termination codon.

**Supplementary Figure S6. Confocal microscopy images of peroxisomes and mitochondria in true leaf epidermal cells**

Fluorescent protein construct visualizing peroxisomes by RFP (magenta) were transiently expressed in true leaf epidermal cells of Arabidopsis at 41 DAS. Mitochondria were simultaneously visualized using mt-GFP. Scale bars = 10 μm.

**Supplementary Figure S7. Mitochondrial response to cold treatment.**

**A)** Representative images of cotyledon epidermal cells at 19 DAS under 22°C (room temperature, RT) and after 1 hour incubation at 4°C (cold treatment, CT). Scale bars = 10 μm. **B)** Quantification of morphology of mitochondria of cotyledon epidermal cells at 15 DAS under RT and CT (white shading bars) conditions (n = 3; error bars = SE). Representative classification images are shown at the bottom right; these images are the same as those presented in Figure 1C. Scale bars = 10 μm. Asterisks indicate significant differences compared to RT (*P* < 0.05).

**Supplementary Table S1**

DRP3A/3B, ELM1/2, FIS1A/1B, and PMD1/2 homologs collected from NCBI database and Phytozome (for liverwort) and used to construct the CLUSTALW sequence arraignment (Supplementary Figure S2).

**Supplementary Table S2**

List of primer pairs used in this study.
